# Forecasting CO_2_ emissions from road fuel combustion using grey prediction models: A novel approach

**DOI:** 10.1016/j.mex.2023.102271

**Published:** 2023-06-28

**Authors:** Flavian Emmanuel Sapnken, Hermann Chopkap Noume, Jean Gaston Tamba

**Affiliations:** a Laboratory of Technologies and Applied Science, IUT Douala, P.O. Box 8698, Douala, Cameroon; b Transports and Applied Logistics Laboratory, University Institute of Technology, University of Douala, P.O. Box 8698, Douala, Cameroon; c Energy Insight-Tomorrow Today, PO Box 2043, Douala, Cameroon; d Laboratory of Energy and Electrical and Electronic Systems, Department of Physics, Faculty of Science, University of Yaoundé I, P.O. Box 812, Yaoundé, Cameroon

**Keywords:** CO_2_ emission forecasting, Grey prediction, Hausdorff derivative, Optimization, *Optimized wavelet transform hausdorff multivariate grey model*

## Abstract

This paper proposes an optimized wavelet transform Hausdorff multivariate grey model (OWTHGM(1,N)) that addresses some of the weaknesses of the conventional GM(1,N) model such as inaccurate prediction and poor stability. Three improvements have been made: First, all inputs are filtered using a wavelet transform; second, a new time response function is established using the Hausdorff derivative; and finally, the use of Rao's algorithm to optimise the model's parameters as well as the ξ-order accumulated value of the observation data described by the Hausdorff derivative. In order to demonstrate the effectiveness of OWTHGM(1,N), it is applied to predict CO_2_ emissions from road fuel combustion. The new model scores 1.27% MAPE and 79.983 RMSE, and is therefore more accurate than competing models. OWTHGM(1,N) could therefore serve a reliable forecasting tool and used to monitor the evolution of CO_2_ emissions in Cameroon. The forecast results also serve as a sound foundation for the formulation of energy consumption strategies and environmental policies.

• Modification, extension and optimization of grey multivariate model is done.

• The model is very generic can be applied to a wide variety of energy sectors.

• OWTHGM(1,N) is a valid forecasting tool that can be used to track CO_2_ emissions.

## Graphical abstract

**Figure:**
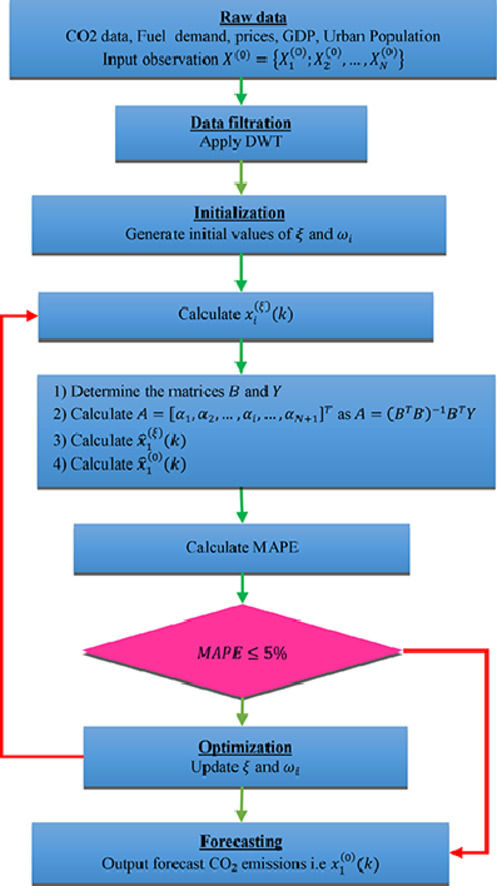

Specifications table

**Table:** 

Subject area:	Environmental Science
more specific subject area:	*modelling and forecasting*
Name of your method:	*Optimized wavelet transform hausdorff multivariate grey model*
Name and reference of original method:	Y. Wang, R. Nie, X. Ma, Z. Liu, P. Chi, W. Wu, B. Guo, X. Yang, L. Zhang, A novel Hausdorff fractional NGMC(p,n) grey prediction model with Grey Wolf Optimizer and its applications in forecasting energy production and conversion of China, Applied Mathematical Modelling. 97 (2021) 381–397. 10.1016/j.apm.2021.03.047. R.V. Rao, R.B. Pawar, Constrained design optimization of selected mechanical system components using Rao algorithms, Applied Soft Computing. 89 (2020) 106,141. 10.1016/j.asoc.2020.106141. Z. Zhang, M.A. Kon, Wavelet matrix operations and quantum transforms, Applied Mathematics and Computation. 428 (2022) 127,179. 10.1016/j.amc.2022.127179.
Resource availability:	*World Bank statistics (*https://data.worldbank.org/*)* *International Energy Agency (*https://www.iea.org/*)* *Ministry of Energy (*http://www.minee.cm/*)*

## Introduction

When predicting the evolution of a system and there is very little information, a major difficulty arises, which is that of accurately extracting the system's characteristic without making too strong assumptions. Deng's grey theory offers a way out [1,2]. However, when conventional multivariate grey models (GM(1,N)) are used, the results are very often inconsistent with reality, as Tien [3] pointed out. One way out is to optimise GM parameters with heuristic optimization procedures [4,5]. In some real-world scenarios, these methods have enhanced the GM(1,N) model's prediction capabilities. Despite these efforts, there is still room to improve its flexibility and forecasting performance, which is summarized as follows:

• As demonstrated by Tien [3], the conventional GM(1,N) model is a factor-based model that reflects the behavioural effects of the driving variables on the system. Unfortunately, the fact that information contained in the set of driving variables is incomplete renders the conventional GM(1,N) model, and even some improved versions, unsuitable for forecasting [6,7].
• The input variables might have irrelevant information (called noise) that corrupts the prediction outcomes [8].
• The least squares method is used to solve grey's difference equation in order to estimate the GM(1,N) model's parameters. However, the first-order differential equation resulting from greys model is used to create the time response function. Although the differential equation and the difference equation approximate each other, they are fundamentally distinct [9]. The GM(1,N) model may become unstable as a result of this discrepancy between parameter estimation and parameter application.

Thus, in order to address the above deficiencies, this paper puts forward a new grey multivariate forecasting model based on the Hausdorff's derivative [10] and optimized by Rao's algorithm (abbreviated OWTHGM(1,N)). In order to demonstrate the efficiency and reliability of OWTHGM(1,N), a practical case is considered, which is the forecasting of the annual electricity consumption.

## Methods

### The conventional GM(1,N) model

The conventional GM(1,N) model [3,11,6,12] can be implemented as follows:

***Step 1: Input raw data***

Assume that the observation or raw data is given by the sequence $X^{\left(0\right)}=\left\{X_{1}^{\left(0\right)};X_{2}^{\left(0\right)},\ldots ,X_{N}^{\left(0\right)}\right\}$ where $X_{1}^{\left(0\right)}$ represents CO_2_ emissions (the dependant variable), and $X_{2}^{\left(0\right)},\ldots ,X_{N}^{\left(0\right)}$ are the independent variables (fuel demand, prices, GDP, urban population and vehicle fleet). Also, $X_{i}^{\left(0\right)}=\left\{x_{i}^{\left(0\right)}\left(1\right),x_{i}^{\left(0\right)}\left(2\right),\ldots ,x_{i}^{\left(0\right)}\left(n\right)\right\},\;i=1,2,\ldots ,N$. $X_{1}^{\left(0\right)}$ should be highly correlated to $X_{2}^{\left(0\right)},\ldots ,X_{N}^{\left(0\right)}$.

***Step 2: Generate accumulated data with 1-AGO***

The first-order accumulation generating operation (1-AGO) of the observation data can be defined by Eqs. (1) and (2):

(1) $$X^{\left(1\right)}=\left\{X_{1}^{\left(1\right)};X_{2}^{\left(1\right)},\ldots ,X_{N}^{\left(1\right)}\right\}$$

where,

$$X_{i}^{\left(1\right)}=\left\{x_{i}^{\left(1\right)}\left(1\right),x_{i}^{\left(1\right)}\left(2\right),\ldots ,x_{i}^{\left(1\right)}\left(n\right)\right\},\;i=1,2,\ldots ,N$$

(2) $$x_{i}^{\left(1\right)}\left(k\right)=\sum_{p=1}^{k}x_{i}^{\left(0\right)}\left(p\right),\;k=1,2,\ldots ,n$$

The superscripts (0) and (1) respectively indicate the original sequences and 1-AGO sequences.

***Step 3: Design mean sequences***

The following definitions (Eq. (3)) represent the mean sequences produced by consecutive terms of $X_{i}^{\left(1\right)}$:

(3) $$Z_{i}^{\left(1\right)}=\left\{z_{i}^{\left(1\right)}\left(2\right),z_{i}^{\left(1\right)}\left(3\right),\ldots ,z_{i}^{\left(1\right)}\left(n\right)\right\},\;i=1,2,\ldots ,N.$$

where:

$$z_{i}^{\left(1\right)}\left(k\right)=0.5\left(x_{i}^{\left(1\right)}\left(k-1\right)+x_{i}^{\left(1\right)}\left(k\right)\right),\;k=2,3,\ldots ,n;\;i=1,2,\ldots ,N$$

***Step 4: Establish grey's differential equation***

The basic GM(1,N) model's differential equation can be written as in Eq. (4):

(4) $$\frac{dx_{1}^{\left(1\right)}\left(t\right)}{dt}+\alpha_{1}x_{1}^{\left(1\right)}\left(t\right)=\alpha_{N+1}+\sum_{i=2}^{N}\alpha_{i}x_{i}^{\left(1\right)}\left(t\right)$$

Consequently, if the matrix $B^{T}B$ is invertible, the parameters ${\left[\alpha_{1},\alpha_{2},\ldots ,\alpha_{i},\ldots ,\alpha_{N+1}\right]}^{T}$ can be calculated using the least-squares approach (Eq. (5)). Which is:

(5) $${\left[\alpha_{1},\alpha_{2},\ldots ,\alpha_{i},\ldots ,\alpha_{N+1}\right]}^{T}={\left(B^{T}B\right)}^{-1}B^{T}Y$$

where the matrices B and Y are given by Eqs. (5.1) and (5.2) respectively,

(5.1) $$B=\left(\begin{array}{ccccc} -z_{1}^{\left(1\right)}\left(2\right) & z_{2}^{\left(1\right)}\left(2\right) & \ldots & z_{N}^{\left(1\right)}\left(2\right) & 1\\ -z_{1}^{\left(1\right)}\left(3\right) & z_{2}^{\left(1\right)}\left(3\right) & \ldots & z_{N}^{\left(1\right)}\left(3\right) & 1\\ \vdots & \vdots & \ddots & \vdots & \;\vdots \\ -z_{1}^{\left(1\right)}\left(n\right) & z_{2}^{\left(1\right)}\left(n\right) & \ldots & z_{N}^{\left(1\right)}\left(n\right) & 1 \end{array}\right)\in R^{\left(n-1\right)\times \left(N+1\right)}$$

(5.2) $$Y=\left(\begin{array}{c} x_{1}^{\left(0\right)}\left(2\right)\\ x_{1}^{\left(0\right)}\left(3\right)\\ \vdots \\ x_{1}^{\left(0\right)}\left(n\right) \end{array}\right)\in R^{\left(n-1\right)\times 1}$$

***Step 5: Solve the system's differential equation***

The initial condition can be used to find the solution to Eq. (5):

(6) $${\hat{x}}_{1}^{\left(1\right)}\left(t=1\right)={\hat{x}}_{1}^{\left(0\right)}\left(1\right)=x_{1}^{\left(0\right)}\left(1\right)$$

The fitting value of $X^{\left(1\right)}$ is obtained by substituting $\alpha_{i}$ into Eqs. (4) and (6). Eq. (7) can be used to determine the fitting value for the original sequence $X_{1}^{\left(0\right)}$.

(7) $${\hat{x}}_{1}^{\left(0\right)}\left(t\right)={\hat{x}}_{1}^{\left(1\right)}\left(t\right)-{\hat{x}}_{1}^{\left(1\right)}\left(t-1\right),\;t=2,3,\ldots ,N$$

### Data filtration using wavelet transform

If there is noise or useless information in the raw data, this will corrupt the GM(1,N) model resulting in poor forecasts [8]. To prevent this, data is cleaned using a Wavelet transform (WT) which is a mathematical function that filters various scale components from a continuous-time signal. Basically, WT is a band-pass filter with its bandwidth scaled to half at each level [13]. The scaling function filters out the lowest point of the transform and allows the entire spectrum to be taken into account. Eq. (8) defines a continuous wavelet transform (CWT) of a signal x(t):

(8) $$CWT_{\varphi }\left(u,v\right)=\frac{1}{\sqrt{\left|u\right|}}\int_{-\infty }^{+\infty }x\left(t\right)\varphi^{*}\left(\frac{t-v}{u}\right)dt$$

where the scale and translation parameters, are denoted by u and v (u,v∈R) respectively.

The discrete wavelet transform (DWT) of a signal $x_{j}$ is calculated using Eq. (9):

(9) $$DWT_{x}\left(p,q\right)=\frac{1}{\sqrt{2^{p}}}\sum_{j}x_{j}\varphi^{*}\left(\frac{j-q}{2^{p}}\right)$$

where q=1,2,…N and p are the sampling time and scale factor respectively. N is the number of samples. The most crucial element of the signal is its low order component. The signal's identity is clearified in this component. The signal's high order component, on the other hand, is a representation of the signal's specifics.

The first step in filtering data is to determine the wavelet to be applied. This depends on the nature of the raw sample data (i.e. the signal). Although there are several criteria for choosing the appropriate wavelet [14], a simple way to do this is to perform a correlation analysis. The waveform of the signal is examined along with the shape of a wavelet (DWT or CWT). If the two match, that particular wavelet is be used as the mother wavelet for the signal. Thus, the choice will vary from one sample data to another.

**Fig. A1:**
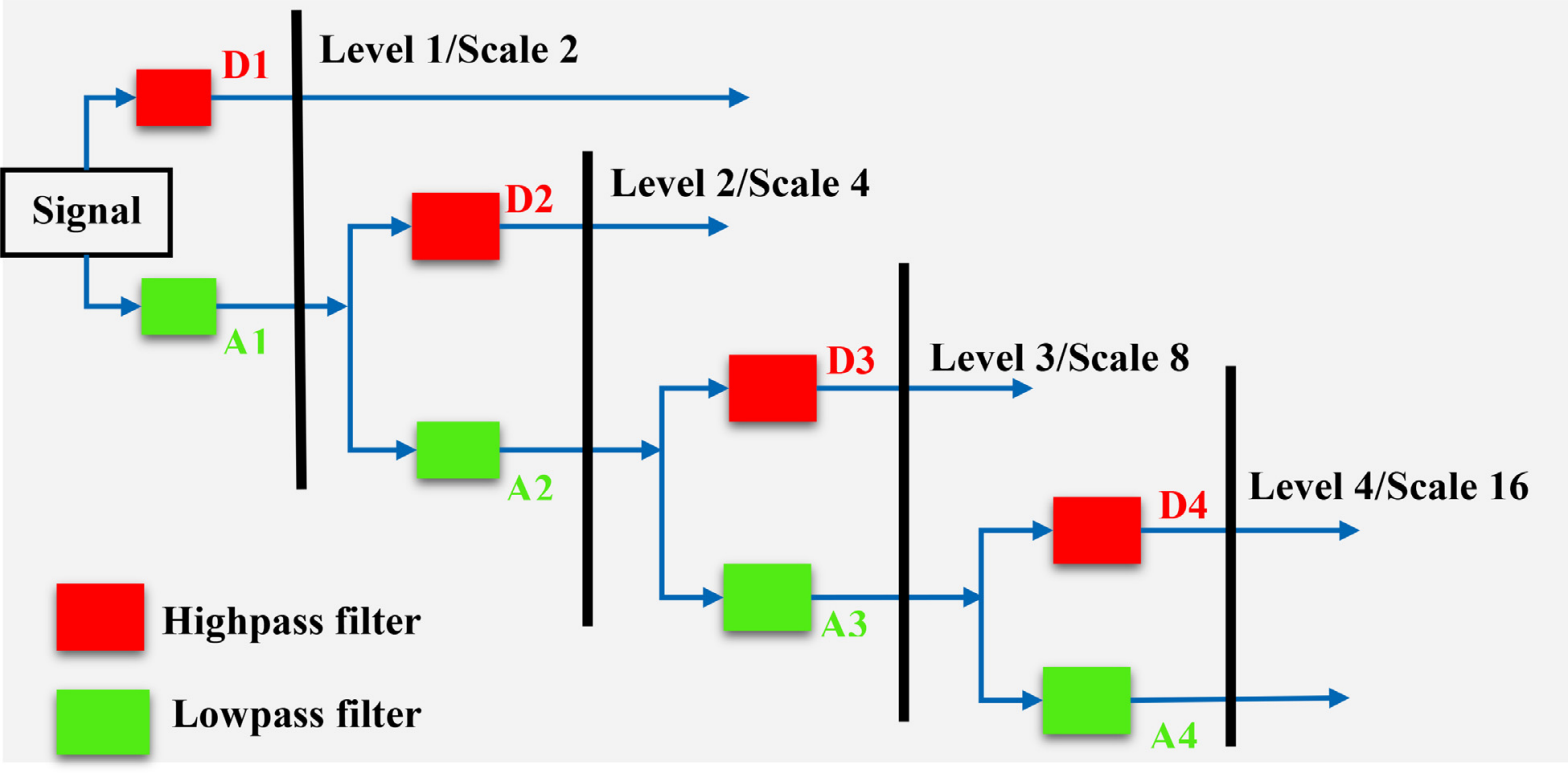
Multilevel wavelet decomposition for a fine scale analysis.

Conceptually, it operates as follows: In order to produce lowpass (A1, generally known as the approximation level) and highpass (D1, also known as the detail level) subbands from a signal X, the signal is first filtered with specialized lowpass and high pass filters (see Fig. A1, in Appendix A). After filtering according to the Nyquist criterion^1^ [15], half of the samples are deleted. The filters often produce strong computational performance and have a limited number of coefficients.

These filters can help eliminate any aliasing brought on by down sampling when reconstructing the subbands. The lowpass subband (A1) is iteratively filtered by the same method to produce narrower subbands (A2, D2, and so on) for the following level of decomposition. Each subband's length of coefficients is divided by the total number of coefficients in the stage before it. In this way, the signal of interest can be captured by a few DWT coefficients of great size, and the signal noise is represented by smaller DWT coefficients. In this manner, DWT aids in the analysis of signals at various resolutions and narrower subbands. Additionally, it aids in signal compression and denoise.

Practically, the overall process of data filtration with DWT is done in Matlab (or other programming languages) based on the following three steps:

***Step 1: Obtain the approximation and detail coefficients.***

To do this, a multilevel wavelet decomposition is used. For a fine scale study, the approximation subband is broken down at several levels or scales (as in Fig. A1).

***Step 2: analyse the details and identify a suitable thresholding technique.***

This is done with Matlab (or other programming languages). Hard thresholding and soft thresholding are the two thresholding operations. The coefficients with magnitudes below the threshold in either operation are set to zero. The way these two methods handle coefficients with magnitudes greater than the threshold is what distinguishes them from one another. In the case of soft thresholding, the coefficients larger than the threshold are kept unaltered, whereas in the case of hard thresholding, they are decreased towards zero by subtracting the threshold value from the coefficient value. In this paper, we used the *sure shrink* with soft thresholding technique to denoise the data.^2^

A single function is used to perform the thresholding of the coefficients as well as the reconstruction of the signal using the new coefficients (see the Matlab code in Appendix B for further information). In this code, *sure shrink* is the thresholding approach. The first parameter ‘f’ refers to the noisy signal. ‘S’ stands for soft thresholding, and the parameter ‘sln’ represents threshold rescaling with a single noise estimate based on first level coefficients. Level indicates the wavelet decomposition level and the last parameter specifies the wavelet, which is *sym6* in this case. The *wden* function decomposes the input signal into many levels, computes and applies a threshold to the detail coefficients, reconstructs the signal using the updated detail coefficients, and outputs it.

***Step 3: Threshold the detail coefficients and reconstruct the signal.***

To begin, use the *wavedec* function to conduct a multilevel wavelet decomposition. The noisy signals are decomposed to five levels. Along with the detail coefficients from the first to the last levels, the function also outputs the fifth level approximation coefficients. The signal's high frequencies are captured by the first level of detail coefficients. The noise in the signal makes up the majority of the high-frequency content, but abrupt signal shifts make up a portion of the high frequency.^3^

The details subband deserves a careful examination. The *detcoef* function is used to extract the coefficients, and the coefficients for each level are plotted. With increasing scale/level, the noise is significantly reduced. If we pay attention to level 1 specifics. The objective in this case is to keep abrupt transitions while removing noise. In order to accomplish this, a threshold is applied to the detail coefficients. The universal threshold is the simplest to compute and is computed using Eq. (10):

(10) $$Universal\;threshold\;=\frac{2\cdot \log\left(length\left(x\right)\right)\cdot median\left(abs\left(D\right)\right)}{0.6745}$$

where x is the signal and D is set of first level detail coefficients.

### The novel Hausdorff GM(1,N) model

***Step 1: Determine the*** ξ ***-order accumulation***

The following introduces the idea of the fractal derivative of a function Ψ(t) with regard to a fractal measure t [10]:

(11.1) $$\frac{d\Psi \left(t\right)}{dt^{\xi }}=\lim_{t^{\prime }\to t}\frac{\Psi \left(t\right)-\Psi \left(t^{\prime }\right)}{t^{\xi }-t^{{}^{\prime }\xi }},\xi >0$$

Eq. (11) is also known as the Hausdorff derivative. $t^{\xi }$ is the fractal time with scale index ξ. If for a given function Ψ(t) both its derivative DΨ(t) and its fractal derivative $D_{\xi }\Psi \left(t\right)$ exists, one can find an analogue to the chain rule:

(11.2) $$\frac{d\Psi \left(t\right)}{dt^{\xi }}=\frac{d\Psi \left(t\right)}{dt}\frac{dt}{dt^{\xi }}=\frac{1}{\xi }t^{\left(1-\xi \right)}\frac{d\Psi \left(t\right)}{dt}$$

Thus, from Eq. (11.2), it is possible to deduce the ξ-order accumulated value of the observation data which then allows to define a new raw data set represented by $X_{i}^{\left(\xi \right)}$ in Eqs. (12) and (13):

(12) $$X_{i}^{\left(\xi \right)}=\left\{x_{i}^{\left(\xi \right)}\left(1\right),x_{i}^{\left(\xi \right)}\left(2\right),\ldots ,x_{i}^{\left(\xi \right)}\left(n\right)\right\},\;i=1,2,\ldots ,N$$

(13) $$\left\{ \begin{array}{c} x_{i}^{\left(\xi \right)}\left(1\right)=x_{i}^{\left(0\right)}\left(1\right)\;\\ x_{i}^{\left(\xi \right)}\left(k\right)=\left(k^{\xi }-{\left(k-1\right)}^{\xi }\right)x_{i}^{\left(0\right)}\left(k\right)+x_{i}^{\left(\xi \right)}\left(k-1\right);\;k=2,3,\ldots ,n;\;i=1,2,\ldots ,N \end{array}\right.$$

***Step 2: Establish grey's differential equation***

The proposed model's differential equation has the following form (Eq. (14)):

(14) $$\frac{dx_{1}^{\left(\xi \right)}\left(t\right)}{dt}+\alpha_{1}x_{1}^{\left(\xi \right)}\left(t\right)=\alpha_{N+1}+\sum_{i=2}^{N}\alpha_{i}x_{i}^{\left(\xi \right)}\left(t\right)$$

$$-\alpha_{1}x_{1}^{\left(\xi \right)}\left(t\right)+\sum_{i=2}^{N}\alpha_{i}x_{i}^{\left(\xi \right)}\left(t\right)+\alpha_{N+1}=\frac{dx_{1}^{\left(\xi \right)}\left(t\right)}{dt}$$

***Step 3: Solve the system's differential equation***

Taking integrals over the interval [k−1,k] on both sides of the above equation, we get Eq. (15):

(15) $$-\alpha_{1}\int_{k-1}^{k}x_{1}^{\left(\xi \right)}\left(t\right)dt+\sum_{i=2}^{N}\alpha_{i}\int_{k-1}^{k}x_{i}^{\left(\xi \right)}\left(t\right)dt+\alpha_{N+1}=\int_{k-1}^{k}dx_{1}^{\left(\xi \right)}\left(t\right)$$

We know that,

$$\int_{k-1}^{k}x_{i}^{\left(\xi \right)}\left(t\right)dt=z_{i}^{\left(\xi \right)}\left(k\right);\;k=2,3,...,n$$

and from Eq. (13)

$$\int_{k-1}^{k}dx_{1}^{\left(\xi \right)}\left(t\right)=x_{1}^{\left(\xi \right)}\left(k\right)-x_{1}^{\left(\xi \right)}\left(k-1\right)=\left(k^{\xi }-{\left(k-1\right)}^{\xi }\right)x_{1}^{\left(0\right)}\left(k\right);\;k=2,3,...,n$$

Therefore, Eq. (15) is as follows:

(16) $$-\alpha_{1}z_{1}^{\left(\xi \right)}\left(k\right)+\sum_{i=2}^{N}\alpha_{i}z_{i}^{\left(\xi \right)}\left(k\right)+\alpha_{N+1}=\left(k^{\xi }-{\left(k-1\right)}^{\xi }\right)x_{1}^{\left(0\right)}\left(k\right);\;k=2,3,...,n$$

Then, Eq. (15) can be written as in Eq. (17.1):

(17.1) BA=Y

Where the matrices A,B and Y are defined as in Eqs. (17.2), (17.3) and (17.4) respectively.

(17.2) $$A=\left(\begin{array}{c} \alpha_{1}\\ \alpha_{2}\\ \begin{array}{c} \vdots \\ \alpha_{N+1} \end{array} \end{array}\right)\in R^{\left(N+1\right)\times \;1}$$

(17.3) $$B=\left(\begin{array}{ccccc} -z_{1}^{\left(\xi \right)}\left(2\right) & z_{2}^{\left(\xi \right)}\left(2\right) & z_{N}^{\left(\xi \right)}\left(2\right) & \ldots & 1\\ -z_{1}^{\left(\xi \right)}\left(3\right) & z_{2}^{\left(\xi \right)}\left(3\right) & \ldots & z_{N}^{\left(\xi \right)}\left(3\right) & 1\\ \vdots & \vdots & \ddots & \vdots & \vdots \\ -z_{1}^{\left(\xi \right)}\left(n\right) & z_{2}^{\left(\xi \right)}\left(n\right) & \ldots & z_{N}^{\left(\xi \right)}\left(n\right) & 1 \end{array}\right)\in R^{\left(n-1\right)\times \left(N+1\right)}$$

(17.4) $$Y=\left(\begin{array}{c} \left(2^{\xi }-1^{\xi }\right)x_{1}^{\left(0\right)}\left(2\right)\\ \left(3^{\xi }-2^{\xi }\right)x_{1}^{\left(0\right)}\left(3\right)\\ \begin{array}{c} \vdots \\ \left(n^{\xi }-{\left(n-1\right)}^{\xi }\right)x_{1}^{\left(0\right)}\left(n\right) \end{array} \end{array}\right)\in R^{\left(n-1\right)\times \;1}$$

If $\text{det}(B^{T}B)\neq 0$, then matrix A can be estimated with Eq. (17.5):

(17.5) $$A={\left(B^{T}B\right)}^{-1}B^{T}Y$$

The solution to Eq. (14) is given by Eq. (18):

(18) $${\hat{x}}_{1}^{\left(\xi \right)}\left(t\right)=x_{1}^{\left(0\right)}\left(1\right)e^{\alpha_{1}\left(1-t\right)}+0.5\sum_{\tau =2}^{t}\left(f\left(\tau \right)e^{\alpha_{1}\left(\tau -t\right)}+f\left(\tau -1\right)e^{\alpha_{1}\left(\tau -t-1\right)}\right);\;t\geq 2$$

where the function f(τ) is expressed as in Eq. (19),

(19) $$f\left(\tau \right)=\alpha_{N+1}+\sum_{i=2}^{N}\alpha_{i}x_{i}^{\left(\xi \right)}\left(\tau \right)$$

***Step 4: Generate forecast values***

Appendix B provides a demonstration of how Eq. (18) is obtained. The following formula can be used to find the fitted value of the original sequence $X_{1}^{\left(0\right)}$:

(20) $$\left\{ \begin{array}{c} {\hat{x}}_{1}^{\left(0\right)}\left(1\right)=x_{1}^{\left(0\right)}\left(1\right)\;\\ {\hat{x}}_{1}^{\left(0\right)}\left(k\right)=\frac{{\hat{x}}_{1}^{\left(\xi \right)}\left(k\right)-{\hat{x}}_{1}^{\left(\xi \right)}\left(k-1\right)}{\left(k^{\xi }-{\left(k-1\right)}^{\xi }\right)};\;k=2,3,\ldots ,n \end{array}\right.$$

Eq. (20) allows to calculate the forecast values. It is known as the wavelet transform Hausdorff grey multivariate model (abbreviated WTHGM(1,N)).

### Optimizing wavelet transform Hausdorff GM(1,N) parameters

Eq. (18) can be expressed using the modified trapezoidal integral formula (given by Eq. (21)):

(21) $${\hat{x}}_{1}^{\left(\xi \right)}\left(t\right)=x_{1}^{\left(0\right)}\left(1\right)e^{\alpha_{1}\left(1-t\right)}+\sum_{\tau =2}^{t}\left(\omega_{1}f\left(\tau \right)e^{\alpha_{1}\left(\tau -t\right)}+\left(1-\omega_{1}\right)f\left(\tau -1\right)e^{\alpha_{1}\left(\tau -t-1\right)}\right)$$

where $\omega_{1}\left(0\leq \omega_{1}\leq 1\right)$ is a parameter.

Still with modified trapezoidal integral formula, Eq. (17.3) can be rewritten as in Eq. (22):

(22) $$B=\left(\begin{array}{ccccc} -{\tilde{z}}_{1}^{\left(\xi \right)}\left(2\right) & {\tilde{z}}_{2}^{\left(\xi \right)}\left(2\right) & \ldots & {\tilde{z}}_{N}^{\left(\xi \right)}\left(2\right) & 1\\ -{\tilde{z}}_{1}^{\left(\xi \right)}\left(3\right) & {\tilde{z}}_{2}^{\left(\xi \right)}\left(3\right) & \ldots & {\tilde{z}}_{N}^{\left(\xi \right)}\left(3\right) & 1\\ \vdots & \vdots & \ddots & \vdots & \vdots \\ -{\tilde{z}}_{1}^{\left(\xi \right)}\left(n\right) & {\tilde{z}}_{2}^{\left(\xi \right)}\left(n\right) & \ldots & {\tilde{z}}_{N}^{\left(\xi \right)}\left(n\right) & 1 \end{array}\right)\in R^{\left(n-1\right)\times \left(N+1\right)}$$

where ${\tilde{z}}_{i}^{\left(\xi \right)}\left(k\right)=\left(\omega_{i+1}x_{i}^{\left(\xi \right)}\left(k-1\right)+\left(1-\omega_{i+1}\right)x_{i}^{\left(\xi \right)}\left(k\right)\right),\;k=2,3,\ldots ,n;\;i=1,2,\ldots ,N$ and $\omega_{i}\;\left(i=2,3,.\;.\;.,\;N+1\right)$ with $\left(0\leq \omega_{i}\leq 1\right)$ represents the coefficient.

An optimization procedure with the mean absolute percentage error (MAPE) set as the objective function can be used to find ξ and $\omega_{i}$. Eq. (23) gives a definition of MAPE.

(23) $$MAPE=\frac{1}{n}\sum_{k=1}^{n}\left|\frac{e_{k}}{x_{1}^{\left(0\right)}\left(k\right)}\right|\cdot 100\%$$

where $e_{k}=x_{1}^{\left(0\right)}\left(k\right)-{\hat{x}}_{1}^{\left(0\right)}\left(k\right)$, and n is the data size. The minimum MAPE value is then determined using a meta-heuristic approach. The ideal values of ξ and $\omega_{i}$ are chosen so as to minimize the difference between the predicted CO_2_ and the emission over a of m years. By solving the following optimization problem (Eqs. (24.1) and (24.2)), the optimal values of ξ and $\omega_{i}$ are ascertained for this purpose:

(24.1) $$minimizeMAPE=\sum_{j=1}^{m}MAPE_{j}$$

(24.2) $$s.t.\;\left\{ \begin{array}{c} 0\leq \omega_{i}\leq 1,\;i=1,2,3,.\;.\;.,\;N+1\\ \xi >0\; \end{array}\right.$$

**Fig. 1:**
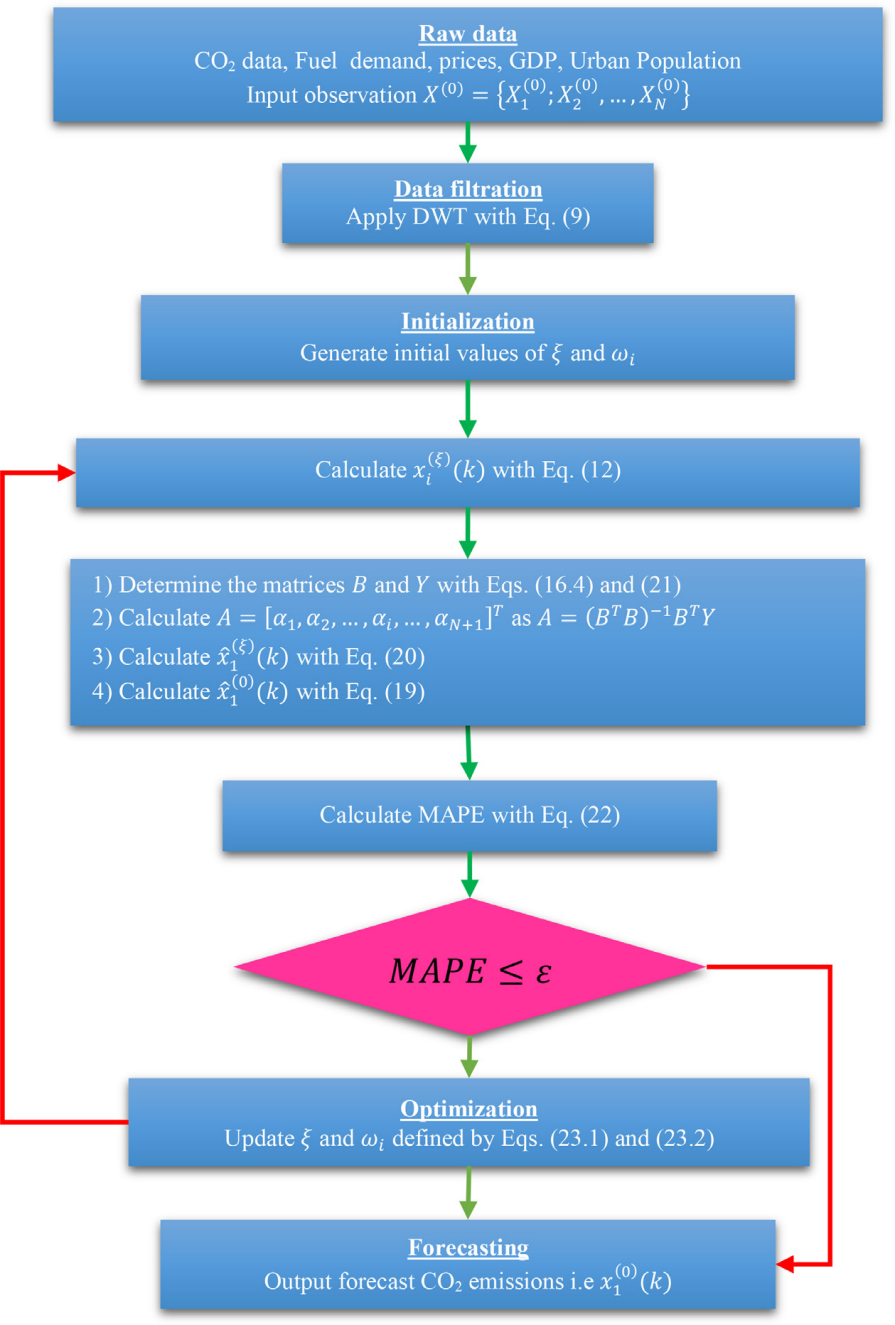
Flow chart of wavelet transform Hausdorff grey multivariate model.

Fig. 1 represents the flowchart of the WTHGM(1,N) model. A meta-heuristic algorithm is employed to solve the suggested optimization problem, as shown on Fig. 1. The condition set on the MAPE depends on the different cases. Thus, the threshold can be set to a certain $\varepsilon =\mathrm{MAP}E_{\min}$, the value of which will be chosen according to the precision that we wish to achieve.

### Selection of meta-heuristic algorithm

Other meta-heuristic algorithms such as genetic algorithms (GA), ABC and particle swarm optimization (PSO) need parameters that are algorithm-specific in addition to usual parameters like iterations and population size. For instance, while PSO needs inertia weight and elements related to social and cognitive learning, GA have special operators such as selection, mutation probability and crossover probability, whereas ABC requires number of employed bees, onlooker bees, scout bees and limits, etc.

Incorrectly setting algorithm-specific parameters can lead to undesired results, such as increasing convergence time or falling into local optimization. However, tuning the specific parameters is a very laborious process. The optimal specific parameters may vary from model to model and may vary from dataset to dataset. The selection of optimal parameters itself is an optimization problem.

Rao's algorithms, which have been developed recently, are straightforward and relatively simple to implement as they do not rely on the use of any algorithm-specific parameters. In particular, the improved Rao algorithm [16] is an algorithm that reinforces both exploitation and exploration, and has a fast convergence speed and a strong ability to deviate from local optimization.

### Improved Rao algorithm for the WTHGM(1,N) model

Here, the population P consists of np individuals $p=\left(\xi ,\omega_{1},\ldots ,\omega_{N+1}\right)\in R^{N+2}$. Initially, each individual is randomly generated while satisfying the constraints of Eq. (24.2). Population updates are carried out on local exploitation phase or global exploration phase with a probability of 0.5 to enhance both exploitation and exploration abilities.

In local exploitation phase, P is sorted in incremental order of MAPE values. The first half population with the smallest MAPE values is considered as the best individuals while the worst individuals is the second half. Following that, Eqs. (25) and (26) are used to compute the local best-mean (${\bar{L}}_{BM}$) and the local worst-mean (${\bar{L}}_{WM}$) vectors respectively:

(25) $${\bar{L}}_{BM}={\bar{M}}_{best}+r_{1}\cdot \left(p_{best}-{\bar{M}}_{best}\right)$$

(26) $${\bar{L}}_{WM}={\bar{M}}_{worst}+r_{2}\cdot \left(p_{worst}-{\bar{M}}_{worst}\right)$$

Here $r_{1}$ and $r_{2}$ are uniformly distributed random vectors in the range ${\left[0,1\right]}^{N+2}$. $p_{best}$ and $p_{worst}$ are the best and worst individuals in P respectively. ${\bar{M}}_{best}$ and ${\bar{M}}_{worst}$ are the mean vectors of the best and worst population respectively. The new individual $p{{}^{\prime }}_{cur}$ is generated with Eq. (27):

(27) $$p{{}^{\prime }}_{cur}=p_{cur}+r_{3}\cdot \left({\bar{L}}_{BM}-{\bar{L}}_{WM}\right)+r_{4}\cdot [\left(p_{cur}\;\text{or}\;p_{sel}\right)-\left(p_{sel}\;\text{or}\;p_{cur}\right)$$

Here $r_{3}$ and $r_{4}$ are uniformly distributed random vectors in the range ${\left[0,1\right]}^{N+2}$. $p_{sel}$ is the randomly selected other individual in P. If $p_{cur}$ is better than $p_{sel}$, $\left(p_{cur}\;\text{or}\;p_{sel}\right)$ means $p_{cur}$, otherwise $p_{sel}$.

In local exploration phase, the global population Q is considered. In the first iteration, Q is set as Q=P and from the second iteration, the new Q is the old Q or the updated P with a probability of 0.5. Then the individuals in Q are shuffled within itself. The new individual $p{{}^{\prime }}_{cur}$ is generated with Eq. (28):

(28) $$p{{}^{\prime }}_{cur}=p_{cur}+rn\cdot \left(q_{cur}-p_{cur}\right)$$

Here $p_{cur}$ and $q_{cur}$ are the cur-th individual in P and Q respectively. rn is the normally distributed random vector in the range ${\left[0,1\right]}^{N+2}$.

After generating new individual $p{{}^{\prime }}_{cur}$, it is compared with the old $p_{cur}$. If $p{{}^{\prime }}_{cur}$ is better than $p_{cur}$, $p_{cur}$ is replaced with $p{{}^{\prime }}_{cur}$ in P. This process is repeated until the MAPE of the best individual $p_{best}$ is smaller than the criterion ($MAPE_{min}$) or until the current number of iterations exceeds the maximum number of iterations ($Iter_{max}$). The algorithmic process of improved Rao algorithm for the WTHGM(1,N) model is shown in the pseudocode below:

**Pseudocode:** Improved Rao algorithm for the WTHGM(1,N) model

**Table:** 

**Input**: dataset for model $X^{\left(0\right)}=\left\{X_{CO_{2}}^{\left(0\right)};{\;X}_{\mathrm{GDP}}^{\left(0\right)};{\;X}_{\mathrm{Urb}}^{\left(0\right)};{\;X}_{\mathrm{Pri}}^{\left(0\right)};{\;X}_{\mathrm{Veh}}^{\left(0\right)};{\;X}_{\mathrm{Fuel}}^{\left(0\right)}\right\}$, parameters for algorithm (np, $\mathrm{MAP}E_{\min}$, $\mathrm{Ite}r_{\max}$) **Output**: optimal values of ξ and $\omega_{i}$	

01: Decompose $X^{\left(0\right)}$ using mdwtdec function of MATLAB 02: Obtain the denoised data $XD^{\left(0\right)}$ using mswden function of MATLAB	/* Data filtration
**03: For**cur **= 1 to**np 04: Randomly generate ξ and $\omega_{i}$ to satisfy Eq. (24.2), and construct cur-th individual $p_{\mathrm{cur}}=\left(\xi ,\omega_{1},\ldots ,\omega_{N+1}\right)$ of the population 05: Calculate $\mathrm{MAP}E_{\mathrm{cur}}$ of $p_{\mathrm{cur}}$ with $XD^{\left(0\right)}$ using Eq. (23) **06: End for** 07: Construct the population $P=\{p_{1},p_{2},\ldots ,p_{\mathrm{np}}\}$ 08: Initialize the global population Q=P 09: Identify the best individual $p_{\mathrm{best}}$ with the smallest MAPE and the worst individual $p_{\mathrm{worst}}$ with the largest MAPE in the population P	/* Population initialization
10: iter=0 11: **While**$\mathrm{MAP}E_{\mathrm{best}}$*>*$\mathrm{MAP}E_{\min}$ 12: Calculate the mean vector ${\bar{M}}_{\mathrm{best}}$ of the best population 13: Calculate the mean vector ${\bar{M}}_{\mathrm{worst}}$ of the worst population 14: **If**rand < 0.5 then Q=P 15: **For**cur = 1 to np 16: **If**rand < 0.5 then /* Local exploitation phase 17: Calculate the local best-mean vector ${\bar{L}}_{\mathrm{BM}}$ using Eq. (A1) 18: Calculate the local worst-mean vector ${\bar{L}}_{\mathrm{WM}}$ using Eq. (A2) 19: Randomly select the other individual $p_{\mathrm{sel}}$ in population P 20: Generate new individual ${p^{\prime }}_{\mathrm{cur}}$ using Eq. (A3) 21: Else/* Global exploration phase 22: Randomly shuffle the individuals of the global population Q 23: Generate new individual ${p^{\prime }}_{\mathrm{cur}}$ using Eq. (A4) 24: **End if** 25: Change ${p^{\prime }}_{\mathrm{cur}}$ to satisfy Eq. (24.2) 26: Calculate $\mathrm{MAP}{E^{\prime }}_{\mathrm{cur}}$ with $XD^{\left(0\right)}$ using Eq. (23) 27: **If**$\mathrm{MAP}{E^{\prime }}_{\mathrm{cur}}$*<*$\mathrm{MAP}E_{\mathrm{cur}}$ then $p_{\mathrm{cur}}={p^{\prime }}_{\mathrm{cur}}$ 28: End for 29: Identify the best individual $p_{\mathrm{best}}$ with the smallest MAPE and the worst individual $p_{\mathrm{worst}}$ with the largest MAPE in the population P 30: iter=iter+1 31: **If**$\mathrm{iter}\geq \mathrm{Ite}r_{\max}$ then break while loop 32: **End while**	/* Population update
33: Output $p_{\mathrm{best}}$	/* Output

## Applications and numerical simulation

### Data selection, filtering and performance measurement

In order to verify the performance of the OWTHGM(1,N) model, we implemented it to forecast CO_2_ emissions from Cameroon's road fuel combustion. In the OWTHGM(1,N) model implemented in this simulation, the filtering technique applied is DWT (i.e. Eq. (9)). This choice follows from the approach explained at the end of the section entitled **«Data filtration using wavelet transform».**

The selected drivers are: CO_2_ emissions, urban population (UP), GDP, road fuel consumption (RFC), fuel prices (PR) and vehicle fleet (VF) [17,18]. Data used in this study cover the period from 1995 to 2020. A prerequisite for good modelling is the selection of appropriate variables, i.e. those that have a significant influence on CO_2_ emissions. So the first step is to ensure that these variables are highly correlated with CO_2_ emissions before including them as independent variables in the forecasting model.

Correlation analysis (presented in Table 1), shows that CO_2_ emissions are significantly correlated with selected variables. Therefore, it can be concluded that they can be used as inputs to model and forecast CO_2_ emissions. However, it is better to filter the data with WT before the forecasting step. This precaution is crucial because the data may have noise that could introduce data matrix ill-conditioning problems in the estimation of grey model parameters.

**Table 1:** Correlation results between the variables used in this study.

	CO_2_	GDP	UP	RFC	PR	VF
CO_2_	1	0.99^a^	0.82^a^	0.94^a^	0.90^b^	0.92^a^
GDP	0.99^a^	1	0.87^b^	0.93^b^	0.93^a^	0.96^a^
UP	0.82^a^	0.87^b^	1	0.70^a^	0.90^a^	0.93^a^
RFC	0.94^a^	0.93^b^	0.70^a^	1	0.85^a^	0.86^a^
PR	0.90^b^	0.93^a^	0.90^a^	0.85^a^	1	0.86^b^
VF	0.92^a^	0.96^a^	0.93^a^	0.86^a^	0.86^b^	1

a *respectively denotes significantivity at 5%, 1% and 0.1% thresholds*.

Forecast accuracies are assessed based on: Root mean square error (RMSE), fitting degree (FD), Absolute percentage error (APE) and mean APE (MAPE), defined in Eqs. (29)–(31):

(29) $$APE=\left|\frac{e_{i}}{x_{1}^{\left(0\right)}\left(i\right)}\right|$$

(30) FD=1−MAPE

(31) $$RMSE=\sqrt{\frac{1}{h-1}\sum_{i=2}^{h}{\left(e_{i}\right)}^{2}}$$

where $e_{i}=x_{1}^{\left(0\right)}\left(i\right)-{\hat{x}}_{1}^{\left(0\right)}\left(i\right)$ represents the i^th^ simulation error between actual CO_2_ emissions $x_{1}^{\left(0\right)}\left(i\right)$ and predicted outcomes ${\hat{x}}_{1}^{\left(0\right)}\left(i\right)$. h is the total number of predictions and ${\bar{x}}_{1}^{\left(0\right)}\left(i\right)$ is the mean of $x_{1}^{\left(0\right)}\left(i\right)$.

Scores of MAPE and RMSE closest to zero indicate the best accuracy. Table 2 shows the threshold values for MAPE. Coming to FD, a score greater than 0.95 indicates a very high precision, and a score between 0.85 and 0.95 indicates a good forecasting accuracy. Overall, the more FD score is close to 1.0, the more accurate the forecasting model is.

**Table 2:** Threshold values for MAPE error metric [19,20].

MAPE(%)	Accuracy level	MAPE(%)	Accuracy level
]0;5]	Class I: Very high precision	]10;20]	Class III: Average precision
]5;10]	Class II: Good precision	]20;+∞[	Class IV: Low precision

The forecasting model may overfit or underfit in case of data leakage. To ensure that this does not happen, the data set is divided into two (training and test sets). These two sets are concealed from each other so that there is no leakage.^4^

### Modelling CO_2_ emissions

Prediction of CO_2_ emissions from Cameroon's road fuel combustion are estimated with WTHGM(1,N) optimized by Rao's algorithm (denoted OWTHGM). For validation purposes, we also compare the estimates with GM(1,N) and WTHGM(1,N) models, as well as multilinear regression (MLR) as applied in the work of Karakurt and Aydin [21]. Training data spans from 1995 to 2017 while test data cover the period or this, the reference and new models are implemented using data collected over the period 1995–2017 to train them (i.e. parameterise the models), while data from 2018 to 2020 are used to test the forecasting performance of each model. In all, 24 simulations were performed on Matlab R2021a using a PC with 8.0 GB RAM. To go systematically, here is how to proceed:

***Stage 1: Determining the filtering technique***

We start by creating a copy of the raw data. One copy is filtered to remove any potential disturbances while the other copy is used as is. As mentioned above, we examined the shape of the wavelet and it matched with the DWT. Thus we apply Eq. (9) to denoise the input data. The next stage is modelling.

***Stage 2: Modelling the WTHGM(1,N)***

At this stage the unfiltered copy is used to establish the WTHGM(1,N) model. To do this, Eqs. (11) to (20) are applied.

***Stage 3: Optimisation of the WTHGM(1,N)***

Here, we use the copy of the raw data that has been filtered and build the OWTHGM(1,N) model given by Eqs. (11) to (28) (see pseudocode). With Rao's algorithm, the population size was fixed at 50 for 10^5^ iterations. In the specific context of our data, we obtained the optimum parameters shown in Table 3:

**Table 3:** Optimum parameters obtained with data on CO_2_ emissions from road transport in Cameroon.

	ξ	$\omega_{1}$	$\omega_{2}$	$\omega_{3}$	$\omega_{4}$	$\omega_{5}$	$\omega_{6}$	$\omega_{7}$
GM(1,N)	1	0.5	0.5	0.5	0.5	0.5	0.5	0.5
WTHGM(1,N)	0.884	0.5	0.5	0.5	0.5	0.5	0.5	0.5
OWTHGM(1,N)	0.148	0.376	0.425	8.9E-4	0.159	3.7E-4	0.026	0.001

***Stage 4: Generating all results***

Finally, we generate all the forecast results given by the WTHGM(1,N) and OTHGM(1,N) models. From these results, we calculate the performance of both models to determine their MAPE, FD and RMSE. This allows us to see which model was more accurate.

**Fig. 2:**
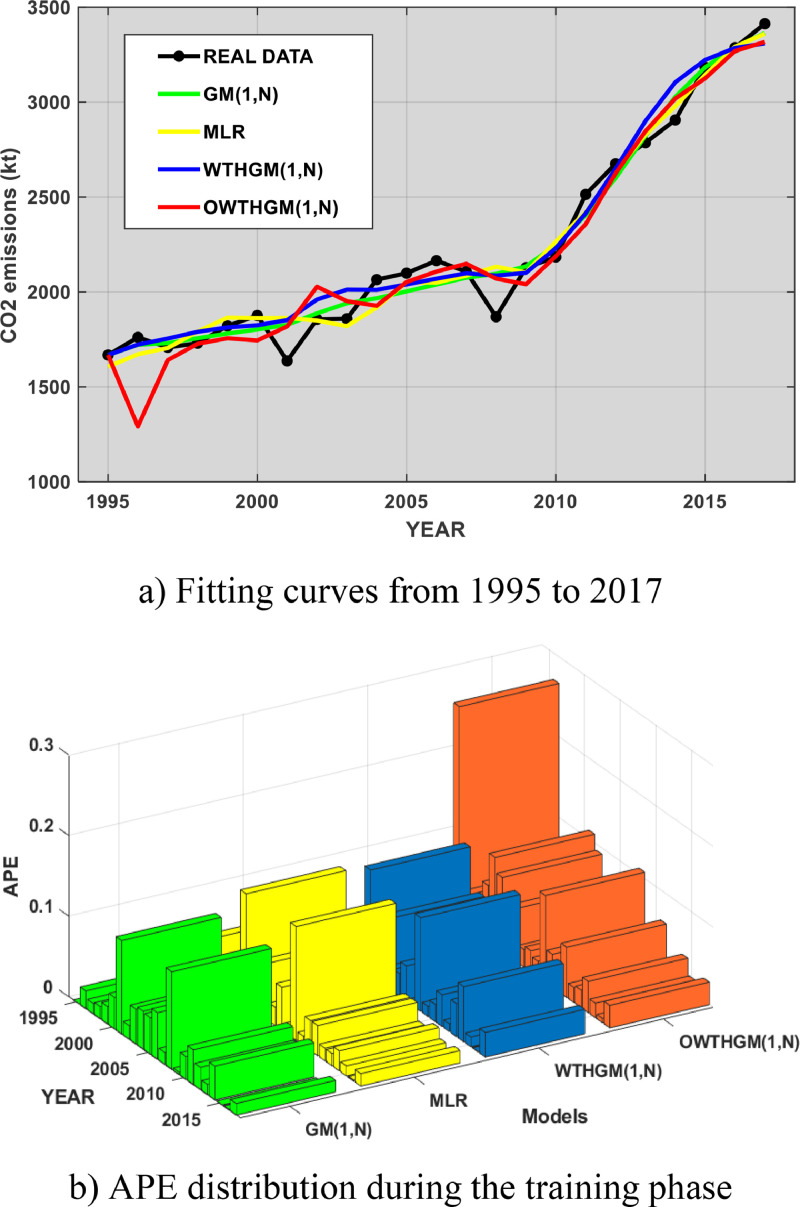
Illustration of the fitting curves and APE during the training phase.

As shown in Table 4, it can be seen that with the training data (1995–2017), the conventional GM(1,N) model, WTHGM(1,N), its optimised version (OWTHGM), and MLR have similar performance in terms of MAPE. They all score a MAPE below 5%, which allows concluding that they behave like class I models according to Table 2. However, we note that the classical GM(1,N), WTHGM(1,N) and MLR models slightly outperform the new model in terms of RMSE, FD and MAPE. Fig. 2a shows the fit curves of the new OWTHGM(1,N) model and the competing models. It is obvious that the OWTHGM(1,N) model (red curve) needs a short fitting time (represented by the first three predictions in the training phase) before it can efficiently extract the systems’ information. This delay is confirmed by its FD, which is only 95% (see Table 4), the lowest of the four models. This explains why in Fig. 2b the peak of APEs is observed on the column of OWTHGM(1,N) model, indicating a lower forecasting accuracy in the training phase.

**Table 4:** Fitted and predicted values of CO_2_ emissions (in kt) with training data.

Year	Real data	GM(1,N)	MLR	WTHGM(1,N)	OWTHGM(1,N)
1995	1668	1668	1606.71	1668	1668
1996	1761	1721.73	1671.70	1722.76	1291.66
1997	1708	1730.46	1704.97	1754.76	1641.11
1998	1730	1756.61	1791.29	1790.21	1727.09
1999	1821	1781.10	1864.94	1813.34	1756.08
2000	1876	1802.23	1862.31	1824.51	1744.61
2001	1637	1827.01	1862.94	1852.04	1818.67
2002	1856	1887.73	1849.80	1960.19	2027.60
2003	1859	1940.24	1820.09	2012.40	1951.53
2004	2065	1967.04	1919.09	2011.08	1927.45
2005	2099	2003.21	2051.08	2039.87	2052.74
2006	2165	2039.29	2049.09	2070.10	2107.53
2007	2107	2075.13	2085.56	2098.51	2148.74
2008	1869	2095.80	2133.20	2086.26	2070.28
2009	2128	2134.90	2103.10	2101.08	2040.63
2010	2184	2246.84	2265.30	2230.97	2188.46
2011	2514	2404.99	2420.01	2414.69	2357.28
2012	2675	2600.24	2633.33	2649.13	2624.88
2013	2786	2822.20	2828.60	2900.19	2846.55
2014	2906	3027.95	2978.35	3104.76	3019.03
2015	3183	3183.27	3143.31	3222.14	3125.21
2016	3287	3289.80	3291.79	3285.38	3269.09
2017	3413	3363.69	3360.44	3309.34	3317.46
MAPE		3.26%	3.43%	3.65%	4.97%
FD		0.97	0.97	0.97	0.95
RMSE		90.61	96.05	102.60	142.67

**Fig. 3:**
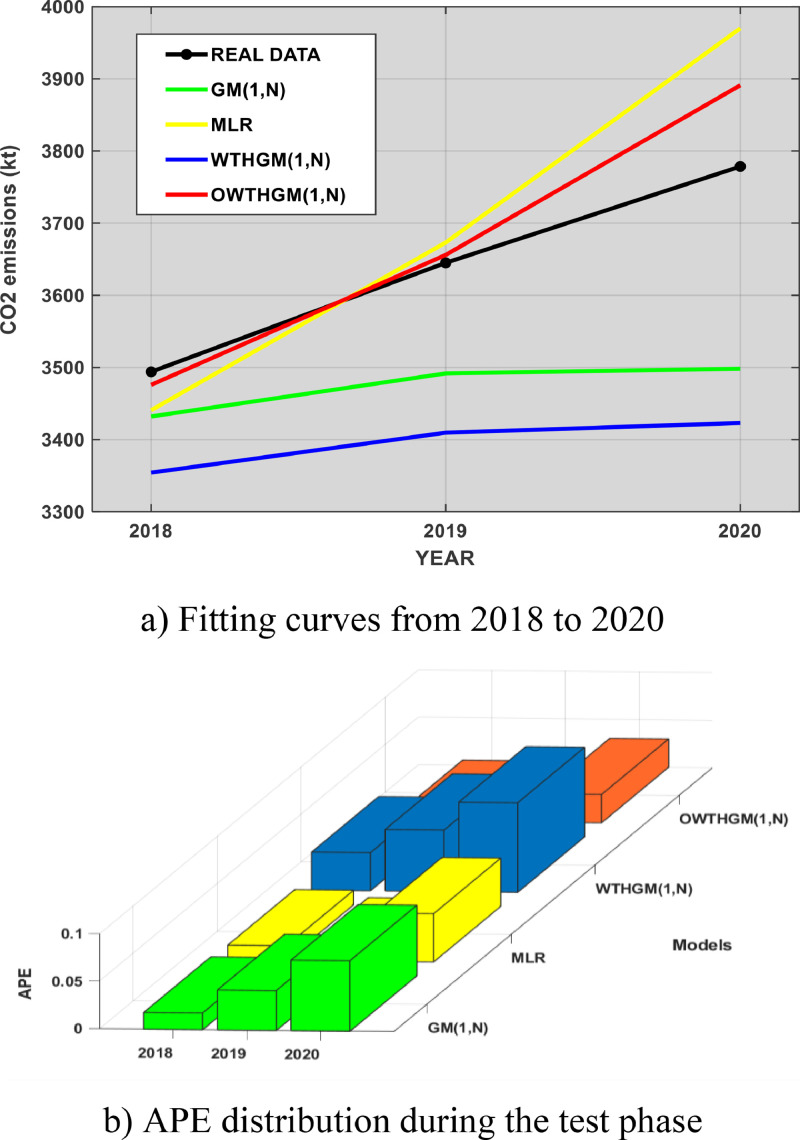
Illustration of the fitting curves and APE during the training phase.

When data is hidden from each model to check whether they are able to predict CO_2_ emissions over the period 2018–2020 (test phase), OWTHGM(1,N) model significantly outperforms the competing models. It appears that only MLR can cope with the novel model. It can be seen in Fig. 3a how the predictive curve of OWTHGM(1,N) comes closest to real data. Moreover, FD criteria shows that OWTHGM(1,N) model has the best fit with a score of 99% while its MAPE and RMSE are the closest to zero with scores of 1.27% and 79.99 respectively (see Table 5). Fig. 3b confirms this performance as the novel model actually has the smallest APE distribution.

**Table 5:** Fitted and predicted values of CO_2_ (in kt) emissions with test data.

Year	Real data	GM(1,N)	MLR	WTHGM(1,N)	OWTHGM(1,N)
2018	3494	3432.09	3440.75	3354.21	3475.86
2019	3645	3491.73	3673.12	3409.59	3655.95
2020	3779	3498.25	3970.02	3423.04	3891.08
MAPE		4.46%	2.45%	6.62%	1.27%
FD		0.95	0.98	0.93	0.99
RMSE		225.863	136.875	301.472	79.983

The model proposed in this paper generates low forecasting errors (less than 5%), leading to the conclusion that OWTHGM(1,N) is a reliable forecasting tool just like its competitors. The novel model can forecast CO_2_ emissions more precisely as well.

As for MLR model, the regression of $X_{1}^{\left(0\right)}$ (i.e. CO_2_ emissions) on independent variables (i.e. $X_{2}^{\left(0\right)},\ldots ,X_{N}^{\left(0\right)}$) reveals that GDP, UP and VF are the most significant determinants of CO_2_ with contributions of 37.1%, 39.8% and 21.6% respectively. R-squared value is 0.97, indicating a strong relationship between CO_2_ emissions and the independent variables. The implication is that the independent variables used in this study manage to explain 97% of Cameroon's road transport CO_2_ emissions. Only 3% of variation remains unexplained, which is certainly due to insufficient data. However, even if more data were collected and other independent variables included, it would never be possible to explain all variations in CO_2_ emissions [22]. Given the performance of OWTHGM(1,N), it can be concluded that the proposed model is better at predicting future CO_2_ emissions without the need to know the underlying functional relationship between the different variables.

In order to strengthen the validation of the new model, we also applied it to other datasets, namely the annual CO_2_ emissions from the Nigerian transport sector obtained from reference [23]. We also divided the data into two groups (training and test sets). The results from the training set are shown in Table 6.

**Table 6:** Adjusted and predicted values of CO_2_ emissions (in Mt) from Nigeria (training data).

Year	Real data	GM(1,N)	MLR	WTHGM(1,N)	OWTHGM(1,N)
1995	15.04	17.35	16.12	14.00	14.95
1996	16.56	15.07	16.48	17.99	17.00
1997	18.45	19.57	18.37	17.24	18.63
1998	16.99	16.97	18.59	17.87	17.01
1999	17.82	19.83	17.80	16.99	17.80
2000	35.47	37.26	35.72	33.97	32.99
2001	39.02	40.02	38.20	41.01	37.91
2002	42.44	44.43	42.90	41.10	42.76
2003	45.00	45.79	42.30	45.15	46.91
2004	44.41	45.08	44.56	43.50	43.99
2005	47.59	46.32	56.67	48.03	47.26
2006	44.01	44.02	48.11	44.99	44.90
2007	42.42	42.23	43.14	40.61	41.71
2008	42.65	42.00	39.21	40.53	41.32
2009	33.70	43.00	35.73	34.03	33.20
2010	50.54	51.25	48.27	50.14	49.01
2011	58.31	57.88	59.33	58.87	58.80
2012	49.08	49.69	47.97	48.74	50.75
2013	52.36	52.12	50.60	51.19	51.98
2014	55.35	54.00	55.00	56.26	55.30
2015	49.15	50.03	48.12	51.96	48.79
2016	52.63	52.02	54.77	50.64	52.30
MAPE		4.70%	4.23%	3.59%	1.86%
FD		0.95	0.96	0.96	0.98
RMSE		2.34	2.62	1.35	0.99

The results shown in Table 6 reveal that the OWTHGM(1,N) model outperforms all competing models on all criteria of comparison. For the forecasts made with the test data (see Table 7), the OWTHGM(1,N) model manages to obtain the best MAPE (1.74%) and therefore the best FD (0.953). When we come to the RMSE criterion, the WTHGM(1,N) model wins with a score of 1.58.

**Table 7:** Adjusted and predicted values of CO_2_ emissions (in Mt) from Nigeria using test data.

Year	Real data	GM(1,N)	MLR	WTHGM(1,N)	OWTHGM(1,N)
2017	50.75	53.72	51.10	49.14	50.02
2018	47.35	47.71	47.98	48.01	48.00
2019	57.29	59.70	54.02	56.10	59.20
2020	58.58	58.56	58.36	57.01	59.00
MAPE		2.71%	1.95%	2.35%	1.74%
FD		0.973	0.980	0.976	0.983
RMSE		1.92	3.13	1.58	1.89

These results and the previous ones allow us to conclude that the new OWTHGM(1,N) model is capable of making very accurate forecasts.

### Forecasting Cameroon's road transport CO_2_ emissions

CO_2_ emissions from the Cameroonian road transport sector over the period 2021–2030 are predicted using the new model proposed in this study and its competitors. In this respect, we used the projected values of the independent variables (RFC, PR, GDP, UP and VF) by a simple regression. These values are confirmed by forecasts of the National Development Strategy [24]. Forecasts results show with great certainty that substantial increases in total CO_2_ emissions from the Cameroonian road transport sector are to be expected in the coming years. In other words, CO_2_ emissions from road transports will increase from 3779 kt in 2020 to 4600 kt in 2030 (see Table 8). Thus, current CO_2_ emissions will increase by 120% in less than ten years. These results are in contrast to the Cameroon government's projections which aim to reduce total CO_2_ emissions by 32% before 2030 [18].

**Table 8:** Projection results of road fuel CO_2_ emissions in Cameroun.

	Models			
Year	GM(1,1)	MLR	WTHGM(1,N)	OWTHGM(1,N)
2021	3548	3563	3490	3832
2022	3668	3650	3606	3819
2023	3789	3736	3717	3896
2024	3912	3822	3824	3992
2025	4036	3908	3929	4093
2026	4161	3995	4032	4194
2027	4286	4081	4133	4295
2028	4413	4167	4234	4397
2029	4540	4253	4333	4498
2030	4668	4339	4432	4600

## Conclusions

This paper proposes an optimized wavelet transform Hausdorff grey multivariate forecasting model (abbreviated OWTHGM(1,N)). In order to validate the model, we implemented it to forecast CO_2_ emissions from road fuel combustion. For this purpose, we started by showing that GDP, urban population, road transport fuel consumption, fuel prices and size of vehicle fleet could be used as determinants of CO_2_ emissions. Forecasts results are compared with the classical GM(1,N), WTHGM(1,N) and MLR, allowing to conclude that:

• GDP, size of vehicle fleet and urban population are the most significant determinants of CO_2_ emissions as confirmed by Refs. [17,18,25]. With these determinants, the proposed OWTHGM(1,N) model manages to completely extract the existing connections they have with CO_2_ emissions.
• Forecasts of CO_2_ emissions produced with OWTHGM(1,N) are more precise than competing models. Thus OWTHGM(1,N) is a reliable tool for monitoring CO_2_ emissions from Cameroon's road transport sector.

These achievements stem from three improvements made in the classical GM(1,N) model. Initially, only significant drivers are considered. Then, all selected drivers are filtered using WT, thereby demonising all series that could hamper modelling. More so, a new time response function has been established using the Hausdorff derivative. Lastly, Rao's algorithm is used to optimise all parameters. The OWTHGM(1,N) model therefore proves to be a reliable forecasting tool and can be used to monitor the evolution of CO_2_ emissions but can also be applied in other forecasting fields characterised by insufficient information.

## CRediT author statement

**Flavian Emmanuel Sapnken:** Conceptualization, Methodology, Software, Writing - original draft preparation. **Hermann Chopkap Noume**: Validity tests, Data curation, Visualization, Investigation. **Jean Gaston Tamba**: Supervision, Validation, Writing-Reviewing and Editing.

## Declaration of Competing Interest

The authors declare that they have no known competing financial interests or personal relationships that could have appeared to influence the work reported in this paper.

## Data Availability

Data will be made available on request.

## Appendix A Multilevel decomposition of the approximation subband

Fig. A1

## Appendix B DWT Matlab code for filtering noisy data

%% Load a signal

%% The entire process should be repeated for each data until all the input data are filtered

load noisysig.mat
figure;
subplot(2,1,1); plot(f0); grid on; title(‘Original signal’); axis tight;
subplot(2,1,2); plot(f); title (‘Original signal with noise’);
axis tight; grid on;

%% Decompose signal using Discrete Wavelet Transform

dwtmode(‘per’,’noisplay’);
wname =’sym6’;
[C, L]=wavedec(f,level,wname);
plotDetCoefHelper(f,C,L);%helperFunction to plot the coefficients at every level

%% analyse the subbands and determine the threshold

%% Denoise the signal

fd=wden(f,’rigrsure’,’s’,’sln’,level,wname);
figure;
subplot(2,1,1);
plot(f); axis tight; grid on; title (‘Noisy Signal’);
subplot(2,1,2);
plot(fd); axis tight; grid on;
title(sprint(‘Denoised Signal SNR;%0.2f dB’,−20*log10(norm(abs(f0-fd))/norm(f0))));

## Appendix B Proof of the derivation of Eq. (17)

From Eq. (13), if we let $f\left(t\right)=\alpha_{N+1}+\sum_{i=2}^{N}\alpha_{i}x_{i}^{\left(\xi \right)}\left(t\right)$, then Eq. (13) can be expressed as:

(A.1) $$\frac{dx_{1}^{\left(\xi \right)}\left(t\right)}{dt}+\alpha_{1}x_{1}^{\left(\xi \right)}\left(t\right)=f\left(t\right)$$

We get the following results by multiplying both sides of Eq. (A.1) by $e^{\alpha_{1}t}$:

(A.2) $$\left(\frac{dx_{1}^{\left(\xi \right)}\left(t\right)}{dt}+\alpha_{1}x_{1}^{\left(\xi \right)}\left(t\right)\right)e^{\alpha_{1}t}=f\left(t\right)e^{\alpha_{1}t}$$

Expressing the left hand side of Eq. (A.2) as a derivative, we get:

(A.3) $$\frac{d}{dt}\left(x_{1}^{\left(\xi \right)}\left(t\right)\cdot e^{\alpha_{1}t}\right)=f\left(t\right)e^{\alpha_{1}t}$$

Taking integrals over the interval [1,t] on both sides of Eq. (A.3) we get:

(A.4) $$\int_{\tau =1}^{\tau =t}d\left(x_{1}^{\left(\xi \right)}\left(\tau \right)\cdot e^{\alpha_{1}\tau }\right)=\int_{\tau =1}^{\tau =t}f\left(\tau \right)e^{\alpha_{1}\tau }d\tau$$

Solving integrals on the left hand side of Eq. (A.4) yields:

(A.5) $$x_{1}^{\left(\xi \right)}\left(t\right)\cdot e^{\alpha_{1}t}-x_{1}^{\left(\xi \right)}\left(1\right)\cdot e^{\alpha_{1}}=\int_{\tau =1}^{\tau =t}f\left(\tau \right)e^{\alpha_{1}\tau }d\tau$$

Multiplying each term of Eq. (A.5) by $e^{-\alpha_{1}t}$ leads to:

(A.6) $$x_{1}^{\left(\xi \right)}\left(t\right)=x_{1}^{\left(\xi \right)}\left(1\right)\cdot e^{\alpha_{1}\left(1-t\right)}+\int_{\tau =1}^{\tau =t}f\left(\tau \right)e^{\alpha_{1}\left(\tau -t\right)}d\tau$$

Recall that $x_{1}^{\left(\xi \right)}\left(t=1\right)=x_{1}^{\left(0\right)}\left(1\right)$, Eq. (A.6) can be rewritten as:

(A.7) $$x_{1}^{\left(\xi \right)}\left(t\right)=x_{1}^{\left(0\right)}\left(1\right)\cdot e^{\alpha_{1}\left(1-t\right)}+\int_{\tau =1}^{\tau =t}f\left(\tau \right)e^{\alpha_{1}\left(\tau -t\right)}d\tau$$

The second term on the right hand side of Eq. (A.7) can be expressed as follows using the trapezoidal integral formula:

(A.8) $$\left\{ \begin{array}{c} \int_{\tau =1}^{\tau =t}f\left(\tau \right)e^{\alpha_{1}\left(\tau -t\right)}d\tau =\sum_{j=2}^{t}\int_{\tau =j-1}^{\tau =j}f\left(\tau \right)e^{\alpha_{1}\left(\tau -t\right)}d\tau \;\\ =\sum_{j=2}^{t}0.5\left(f\left(j\right)e^{\alpha_{1}\left(j-t\right)}+f\left(j-1\right)e^{\alpha_{1}\left(j-t-1\right)}\right) \end{array}\right.$$

When we substitute Eq. (A.8) in Eq. (A.7), we obtain:

(A.9) $${\hat{x}}_{1}^{\left(\xi \right)}\left(t\right)=x_{1}^{\left(0\right)}\left(1\right)e^{\alpha_{1}\left(1-t\right)}+0.5\sum_{\tau =2}^{t}\left(f\left(\tau \right)e^{\alpha_{1}\left(\tau -t\right)}+f\left(\tau -1\right)e^{\alpha_{1}\left(\tau -t-1\right)}\right)$$

## Footnotes

1 In order not to lose the information in the signal, the Nyquist criterion specifies that the sampling frequency must be at least twice as high as the highest frequency present in the signal. An aliasing phenomenon occurs if the sampling frequency is less than twice the maximum frequency of the analogue signal.

2 Soft thresholding is always a good starting point if in doubt about which technique to choose.

3 There are times when these abrupt changes carry meaning, and it might be desirable to retain this information while denoising the signal.

4 Performance problems with predictive models are mainly due to over-fitting or under-fitting. Therefore, splitting the data in two, as suggested, allows testing the generalization of the predictive model on data that it has not yet encountered during training stage.
